# Development of Electrically Conductive Wood-Based Panels for Sensor Applications

**DOI:** 10.3390/polym16213026

**Published:** 2024-10-28

**Authors:** Ozden Beste Kocoglu, Claudia Pretschuh, Christoph Unterweger, Mehmet Kodal, Guralp Ozkoc

**Affiliations:** 1 Polymer Science and Technology Graduate Programme, Kocaeli University, 41001 Kocaeli, Türkiye; b.kocoglu@wood-kplus.at; 2 Wood K Plus—Kompetenzzentrum Holz, 4040 Linz, Austria; c.pretschuh@wood-kplus.at (C.P.); c.unterweger@wood-kplus.at (C.U.); 3 Chemical Engineering Department, Kocaeli University, 41001 Kocaeli, Türkiye; 4 SUNUM, Nanotechnology Research and Application Center, Sabancı University, 34956 Istanbul, Türkiye; 5 Department of Chemistry, Istinye University, 34396 Istanbul, Türkiye

**Keywords:** wood-based composites, sensor, electrically conductive board, carbon fillers

## Abstract

This study investigates the development of electrically conductive panels for application as emergency detection sensors in smart house systems. These panels, composed of wood chips coated with polymeric methylene diphenyl isocyanate, were modified with carbon black and carbon fibers to enable detection of moisture, temperature, and pressure variations. Manufactured via hot pressing, the panels retained standard mechanical properties and exhibited stable performance under diverse environmental conditions. Carbon black-filled panels achieved electrical percolation at a lower filler concentration (5%) compared to carbon fiber-filled panels. The incorporation of carbon black reduced the electrical resistivity to 8.6 ohm·cm, while the addition of carbon fibers further decreased it to 7.7 ohm·cm. In terms of sensor capabilities, panels containing carbon fibers demonstrated superior sensitivity to moisture and pressure changes. However, carbon black was ineffective for temperature sensing. Among the carbon fiber-filled panels, those with 20 wt.% concentration exhibited the best performance for moisture and pressure detection, whereas panels with 40 wt.% carbon fiber content displayed the most reliable and consistent temperature-sensing properties.

## 1. Introduction

With the development of information and communication technologies, communication networks, such as the “Internet of Things (IoT)”, where physical objects connect with larger systems, have attracted great interest from researchers and practitioners in recent years [1]. As the technology in this system continues to progress swiftly, there is also a concurrent development of “smart house” concepts aimed at enhancing the quality of life across various domains.

The evolution of the IoT is reshaping living and working spaces into intelligent environments where a smart house must interact with diverse sensors and actuators in the user’s surroundings to anticipate their requirements. Given the significant role of wood products as building materials, it is essential to align them with advanced technologies and enhance their functionality while preserving their inherent qualities. While the integration of electronic devices into both interior and exterior design is of increasing interest to architects, meeting this challenge requires in addition the need for wireless connectivity technologies in wood-based paneling that allow for adaptive placement and arrangement [2,3]. Wood-based panels can serve as temperature or moisture sensors when used outside buildings and as temperature, moisture, and pressure sensors when employed as wall panels or flooring inside buildings. This multifunctionality enhances their potential applications in smart house systems, contributing to improved environmental monitoring and energy efficiency.

Wood, a renewable and biodegradable resource that naturally stores CO_2_, stands out as a state-of-the-art building material with outstanding aesthetic and textural properties [3]. It is both lightweight and has high mechanical strength. However, the advancement of wood electronics has been constrained by its complex structure and inherent nature as an electrical insulator [4].

Wood-based composites must be made electrically conductive to be used directly as sensor substrates in these technologies. Accordingly, various approaches have been explored in recent years to enhance the electrical properties of wood-based composites. The oldest method involves inducing carbonization of the wooden part directly through pyrolysis to confer electrical conductivity [5,6,7,8]. The literature also mentions the use of metal wires [9,10,11] or metal salt impregnations as a surface coating [12], conductive carbon inks [13], or impregnation with polyaniline [14] to impart electrical conductivity to wood. In addition, numerous publications have demonstrated the application of carbon nanotubes (CNTs) [15,16,17,18], graphene [17], and carbon fibers (CFs) [19,20,21,22] as conductive fillers in wood-based composites or papers to enhance mechanical properties, electromagnetic shielding, thermal conductivity, and electrical conductivity. A conductive coating or layer that can be smoothly integrated into the composite structure is required to achieve conductive wood-based composites. Zhu et al. [19] presented CF–wood composites with a negative temperature coefficient of resistance (NCR). The authors found that the data of surface resistivity represented a negative growth situation with increasing temperature from 20 °C to 120 °C. Depending on the formulation, the resistivity decreased from a range of 5 to 16 ohm.cm to 1 to 3 ohm.cm. Hou et al. [20] indicated that a higher content of shorter CFs was necessary to achieve percolation effects. Tschannen et al. [22] showed the advantage of wet mixed fibers. Panels produced with UF resin showed conductivity values of around 25–230 S/m, while panels produced with polymeric methylene diphenyl isocyanate (pMDI) showed conductivity values around 48–190 S/m. Although conductive carbon black (CB) was not used as a conductive filler for wood-based panels so far, conductive carbon fillers are well-known for their use in wood–plastic composites (WPCs). In some studies, WPCs have been developed using CB nanoparticles and mechanical properties [23,24,25], thermal stability [23], and electrical conductivity [23,26], and high electromagnetic interference (EMI) shielding capabilities have been achieved in the presence of CB [23,24,25,26].

The addition of carbon-based materials, such as carbon nanotubes (CNTs), graphene, carbon fibers (CFs), and carbon black (CB), enhances the electrical conductivity of wood-based panels. As these conductive fillers are dispersed into the insulating wood matrix, a continuous conductive network forms once the percolation threshold is reached. This threshold is influenced by factors like filler concentration, dispersion, and aspect ratio, with materials like CNTs and graphene forming conductive paths even at low concentrations due to their high aspect ratios. The type of wood panel and method of integration further affect conductivity [27,28].

As a general remark, electrical conductivity constitutes an interesting topic with even more wide-ranging applications. It has facilitated the creation of various electrical and electronic components, including sensors [29,30,31], heaters [32,33,34], and shielding mechanisms [35].

In the present work, for the first time, the addition of conductive CB to pMDI-bonded wood-based panels was given and compared directly to the addition of CFs. Their effect on composite homogeneity, percolation threshold, and their applicability for moisture, temperature, or pressure monitoring was determined. For this purpose, conductive particle boards (PBs) were obtained by mixing wood chips with CFs or CB at different ratios. The electrical, mechanical, physical, and morphological properties of the PBs were investigated. Electrical resistivity measurements were taken from the surface and cross-sectional areas of the samples, and the distribution of carbon materials in the PB was also observed. The temperature, moisture, and pressure sensor properties of these high-strength hybrid composites are investigated. Compared to high-cost carbon materials such as single and multi-walled CNTs and exfoliated graphene, high electrical conductivity, lightweight and more cost-effective CB, and CFs were preferred.

In the literature, the electrical conductivity and sensor applications of materials have been widely explored across various fields. Wood-based panels play a particularly important role within the “smart house” concept, yet there are relatively few studies that focus specifically on these materials [2,19,20,21,22]. Most existing research has been centered on improving the electrical conductivity of wood-based panels, without examining their potential for use as sensors. This study addresses that gap by not only enhancing the electrical conductivity of wood-based panels but also investigating their sensor capabilities. Additionally, efforts were made to directly integrate both electrical conductivity and sensor functions into a particle board.

## 2. Materials and Methods

### 2.1. Materials

Wood chips were obtained from Kastamonu Entegre Ağaç Sanayi, Gebze, Turkiye. The wood chips consisted of 70 wt.% pine and 30 wt.% oak. Particle board samples were produced as single layers using the surface layer (SL) formulation. The average size of wood chips was measured by Kastamonu Entegre and reported as 0.7 mm.

The polymeric methylene diphenyl isocyanate (pMDI, Lupranat M 20 S) used in this study was selected according to its use in wood-based panels and obtained from BASF, Lemförde, Germany. It contains high-functionality oligomers and isomers. The used pMDI had a viscosity of 210 mPas at room temperature, a density of 1.24 g/cm³, and an isocyanate content of 32%.

Conductive CB (Vulcan XC 72 R) was sourced from Cabot, Billarica, MA, USA. The producer reports CB to have 96% carbon content and an average size of 50 nm. The BET surface area of CB was measured as 240 m²/g, and the main pore size was in the diameter range of 12–26 nm.

CFs were purchased from Dost Kimya, a local supplier in Istanbul, Turkiye. The supplier reported that the CFs were 97% carbon, with fiber lengths ranging between 100 and 400 µm and a density of 1.84 g/cm^3^. The BET surface area of CFs was measured as 14 m^2^/g, and the main pore diameters were between 2 and 6 nm and 12 and 26 nm. The average single fiber diameter observed from SEM images was approximately 8 µm.

### 2.2. Manufacturing Conductive Particle Board

In this study, laboratory-scale particle board production with a targeted thickness of 8 mm and a density of 700 kg/m^3^ was carried out. PBs were produced according to EN 312 standard [36]. The resin content (pMDI) for boards was kept constant in all productions and was 10% by weight based on dry wood chip mass.

**Figure 1:**
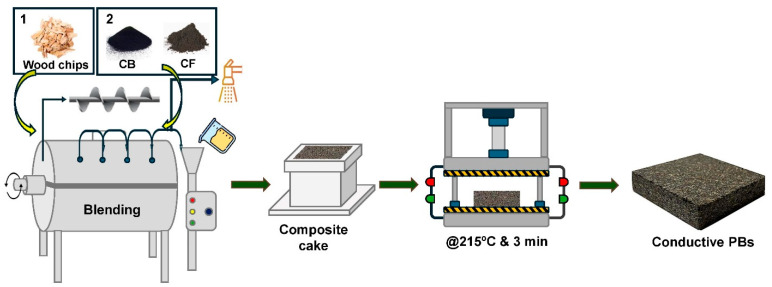
Stages of particle board manufacturing.

In the first step, wood chips were added to the rotary mixer, and resin was sprayed onto the wood chips using a spraying system, see Figure 1. The wood chips and resin were mixed with a rotary mixer operating at a screw speed of 100 rpm. The materials were allowed to mix for 120 s. The resin ratio was kept constant throughout all productions.

In the second stage, conductive carbon material was added to the wood chip-resin mixture at specified rates and mixed at 100 rpm for 120 s. The mixture was then transferred into a 20 cm by 20 cm mold, forming a composite cake. To obtain the final composite product, the composite cake was subjected to a curing process using a hot press (Burkle GmbH, Bad Bellingen, Germany) operating at 215 °C for a period of 180 s and a maximum of 80 bar for all boards. This curing step is crucial in hardening the composite material and improving its overall structural integrity and properties.

The amount of CB and CF additives was calculated relative to the total weight of the wood-based composite. In addition, while determining the additive ratios for each additive material, processability, final composite mechanical, and electrical properties were taken into consideration. For CB additive, it was observed that beyond 20 wt.% of CB, challenges in mixing, inhomogeneous distribution of CB, and reduced mechanical properties of the composite were obtained. Furthermore, it was found that the electrical resistivity of the composites exceeded 1 MΩ when the CF additive was below 20 wt.%. Consequently, this study determined the concentrations of CB and CF additives to be investigated as 5–10–20 wt.% for CB and 20–40–60 wt.% for CF, as shown in Table 1.

Preliminary studies were conducted to determine the optimal press conditions and resin quantity for this study. These conditions were established by examining the mechanical properties of the PBs. Initially, the optimal amount of resin was identified by keeping the press parameters constant to achieve the best mechanical properties. Subsequently, the press time, temperature, and pressure parameters were analyzed to enhance the mechanical properties further. The mechanical properties of the resulting PBs were evaluated, leading to the determination of the optimal pressing conditions and resin quantity.

### 2.3. Characterization

The conductivity (σ) is calculated using the formula σ = L/RA, where L represents the length, R denotes the resistance, and A is the cross-sectional area of the materials. In this study, conductivity analyses were conducted through resistance measurements of the PBs, and the resistance measurements were performed using a Keithley 2110 multimeter with two-probe. Before measurements, copper cables were fixed on top and bottom surfaces of the samples by using RS Pro silver conductive paint. Electrical resistivity measurements were carried out from the surfaces and cross-sections of the samples.

For electrical resistivity measurements, 40 mm × 40 mm samples were cut from the produced PBs. Subsequently, the cables were fixed to two points on both the upper and lower surfaces of the samples, with a 20 mm distance between them, via silver conductive paint. The samples were kept at 25 °C and 50% relative moisture for 48 h, after which measurements were conducted both on the surfaces and the cross-sections.

**Figure 2:**
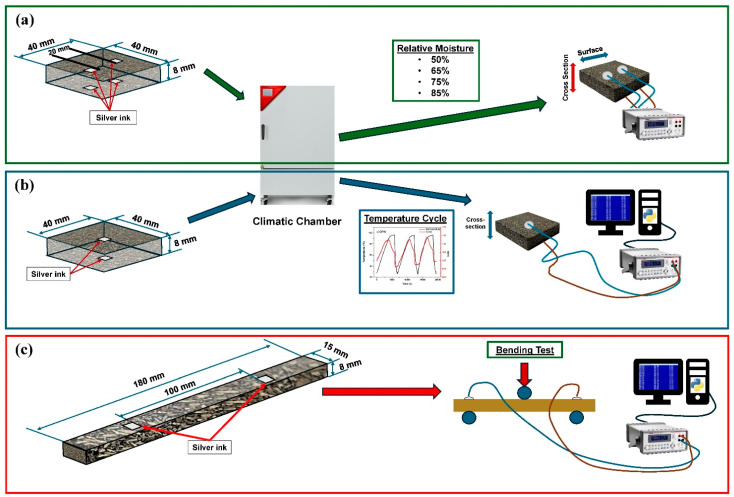
Schematic representation of the characterizations of the sensor properties of particle boards; (**a**) moisture effect; (**b**) temperature effect; (**c**) pressure effect.

In this study, the effects of external factors such as temperature, moisture, and pressure as well as effects from material content on the electrical resistance were investigated (Figure 2). The electrical resistance change of the samples at different moisture levels was determined in a climatic chamber (Figure 2a). The samples were kept at 25 °C and at varied relative moisture, 65–75–85%, for 48 h, and the electrical resistance measurements were examined from surface and cross-section at the end of each 48 h.

The temperature effect on the electrical resistivity was observed with the help of Keithley 2110 multimeter and a K-type thermocouple (Figure 2b). In this study as well as in the moisture study, 40 mm × 40 mm samples were used; measurements were made exclusively in the cross-section direction. The samples were heated and cooled from 25 to 100 °C at a rate of 1 °C/min for three cycles in a climatic chamber at 50% relative moisture. During this process, the change of electrical resistance with temperature was monitored by using the Python 3.12.0 software. The relative resistance is calculated as R/R_0_. R_0_ is the initial resistance value.

With the 3-point bending test, the resistance changes of the samples with increasing pressure were examined (Figure 2c). Measurements were taken between two points, 100 mm apart from the upper surfaces of samples, with sample dimensions of 15 mm × 8 mm × 180 mm. The 3-point bending test was performed with a Zwick Roell Beta 50, Ulm, Germany. During this process, the force and electrical resistance change were monitored using the Python 3.12.0 software. The relative resistance is calculated as R/R_0_. R_0_ is the initial resistance value.

The testing of the internal bond strength (IB) of the composites was performed using a mechanical testing device (ZwickRoell Z010, Ulm, Germany) according to EN 319 standard. The average value of 10 samples was taken.

The morphological characterization of CB and CFs and their distribution in the conductive PBs were investigated by SEM and optical microscopy. SEM analysis was performed on a JEOL JIB-4601 MultiBeam FIB-SEM (Tokyo, Japan) instrument with 2 mm × 2 mm PB samples and imaged at different magnifications. Samples were placed in a vacuum oven at 120 °C for 72 h. The dimensional analysis of CB and CFs was performed using a Thermo Fisher Phenom Pro X SEM (Linz, Austria) device. Optical microscopy analysis was carried out with an Olympus BX-RLA2 (Tokyo, Japan) reflected light microscope.

The specific surface area of the samples was measured through physisorption analysis using an automatic volumetric sorption analyzer (Autosorb-iQ, Anton Paar QuantaTec Inc. Graz, Austria), with nitrogen as the adsorbate at 77 K. Prior to measurement, 50–100 mg of the samples were outgassed for 5 h at 300 °C to remove any contaminants. The adsorbed gas volume was then measured at relative pressures (P/P_0_) ranging from 0.1 to 0.9. The specific surface areas were calculated using the Brunauer–Emmett–Teller (BET) method.

## 3. Results and Discussion

### 3.1. Morphology and Carbon Filler Distribution

Size distribution and morphology images of carbon fillers, as well as the detection of the conductive PBs’ homogeneity and the carbon fillers’ distribution, were obtained using SEM and optical microscopy.

**Figure 3:**
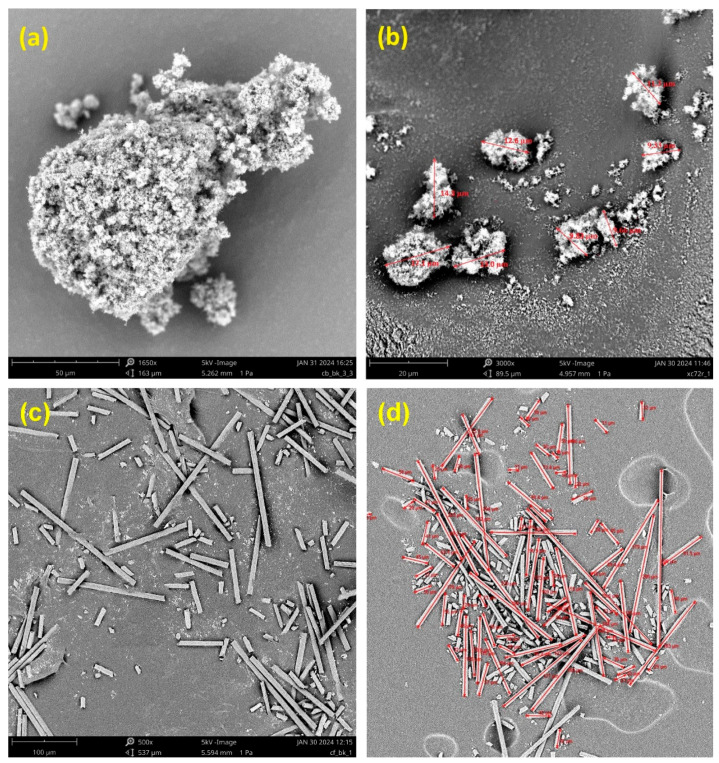
(**a**) SEM images of carbon black agglomerates of carbon black, and (**b**) the sizes of carbon black clusters (**c**) SEM images of carbon fibers, and (**d**) measurements of carbon fiber length.

As presented by SEM images in Figure 3, the used CB particles tend to aggregate into several larger clusters. This results from the interaction between carbon particles under the influence of Van der Waals and electrostatic forces [37,38]. The CB particles possessed a rough surface and, consequently, a larger surface area [39], potentially facilitating the formation of stronger adhesion with the wood matrix [24]. The average size of the clusters was determined to be between 5 and 150 µm (Figure 3a,b).

CFs were observed to have a diameter of 8 µm and an average length of the fibers between 10 and 320 µm (Figure 3c,d).

**Figure 4:**
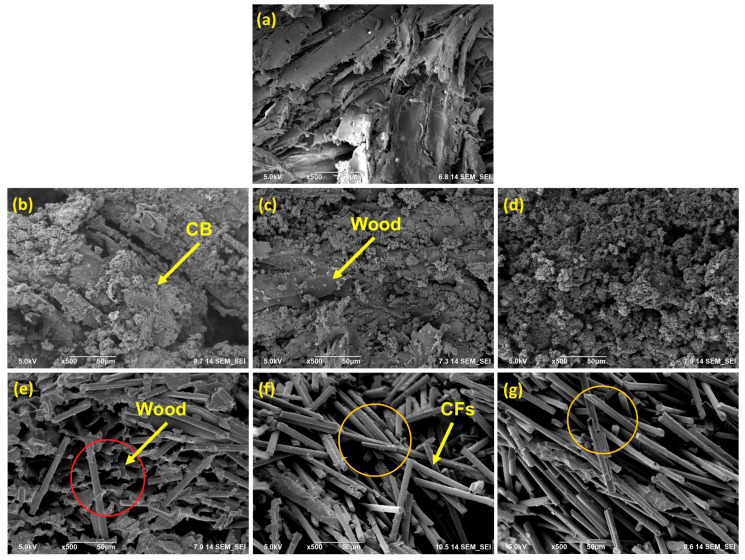
SEM images of (**a**) reference sample; (**b**) CB5; (**c**) CB10; (**d**) CB20; (**e**) CF20; (**f**) CF40; and (**g**) CF60.

CB agglomerates in the wooden matrix are present in SEM images (Figure 4b–d). The average size of such CB agglomerates cannot be readily determined from the SEM images. Although individual CB particles are nanofillers, the overall microstructure of wood–CB composites can be considered as a macro-composite due to the average agglomerate size being larger than 100 nm. The CB conductive agglomerates are well-dispersed throughout the wood matrix in the prepared macro-composite, creating continuous conductive pathways. This effective dispersion and formation of conductive networks ensures that the composite achieves high electrical conductivity even at low filler contents, as will be discussed in the following section.

Figure 4e–g illustrates the irregular and, in contrast to CB, poor dispersion of CFs within the wooden matrix.

Especially by the comparison of both samples prepared with 20 wt.% of filler content, the differences in the distributions between CFs and CB are apparent. In the case of the CB sample, the whole wooden part is covered by the rough surface of the CB particles; for the CF sample, there are single and not-connected fibers present, and many parts of the pure wood are visible. In Figure 4e (CF20), also large voids between the filler and the wooden matrix are visible, highlighted with a red circle, indicating the poor distribution of the CFs in the resin containing wood chips and potentially weak interfacial interaction between the wood and CFs. This poor interaction may result from incompatibility between the CFs and wood. CFs exhibit smooth surfaces and are usually coated with a special surface finish, in contrast to the rough CB agglomerates; this could be the reason for their different interfacial behavior within the pMDI-coated wood phase.

At high CF contents, single and multiple contacts between the CFs were observed in the CF40 and CF60 samples (Figure 4f,g; marked with yellow circulars), suggesting that such contacts are majorly liable for the electrical conductivity throughout the composite structure [38].

**Figure 5:**
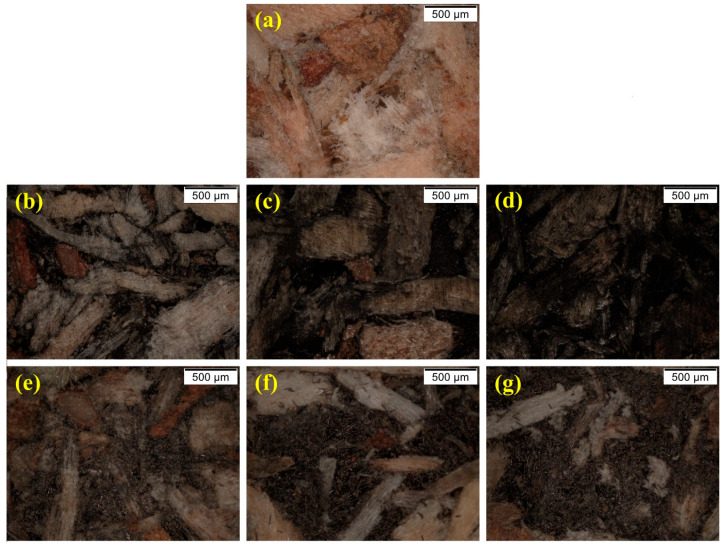
Optical microscopy images of (**a**) reference sample; (**b**) CB5; (**c**) CB10; (**d**) CB20; (**e**) CF20, (**f**) CF40 and (**g**) CF60.

In addition, the optical microscope images in Figure 5 prove the phenomenon of the worse distribution of the CFs in the wooden matrix. The sample CB20 appears black (Figure 5d), indicating the continuous CB network; however, the sample CF20 (Figure 5e) remains brighter and does not cover all the wooden parts. Even for the sample with 60 wt.% CF (Figure 5g) content, bright chips are visible, indicating the more inhomogeneous distribution of the CFs in comparison. As a result, the absence of continuous conductive pathways in the wood–CFs composites led to lower electrical conductivity and therefore higher percolation threshold, as confirmed by conductivity measurements in the next section.

Pourjafar et al. [21] mentioned that CFs became better visible in the wood fiber matrix if the CFs content is increased to 10 wt.%. This fits with our investigation that 20 wt.% of CFs are needed to form a sufficient network (see also Figure 5). This also agrees with Hou et al. [20], where a 3-dimensional conductive network was seen as the CFs content was increased to 20 wt.%; however, in this study, long CFs of a minimum of 2 mm length have been used. Zhu and Sun [19] even applied a high amount of 55 wt.% of CFs, in this case, short-cut fibers, to prepare a conductive network on the surface.

### 3.2. Electrical Properties

**Figure 6:**
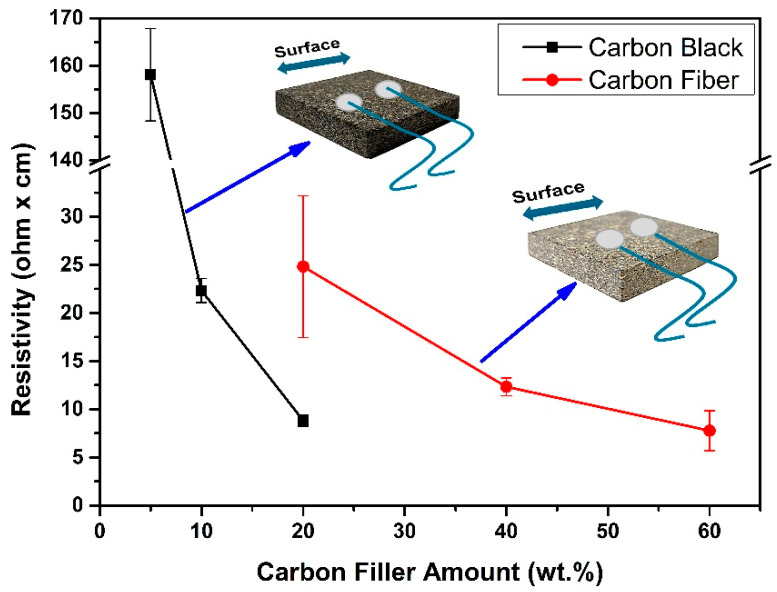
Surface electrical resistivity of conductive particle boards.

Figure 6 shows the average electrical resistance values. Measurements were made on the surface of the sample, and an average of ten measurements of five different samples had been taken. It was observed that the electrical resistance values decreased, as the proportion of carbon-based additives increased.

The incorporation of CB substantially enhanced the electrical conductivity of the PBs, even at low loadings, in contrast to the CF composites. This distinct behavior, compared to the CF composites, can be attributed to the smaller average particle size and its spherical shape and the higher surface area of CB leading to the observed better dispersion properties of CB. For a better-dispersed additive, there is an increasing likelihood of contact points and a greater propensity to form a continuous conductive network within the wooden composite [40,41,42,43].

The higher surface area of the CB increases the dispersion and interaction within the wooden matrix, and hence, its conductivity increases, leading to a lower percolation threshold [44]. Highly structured CB aggregates develop numerous conductive pathways at the nanoscale, in contrast to the larger, micron-sized CF. This characteristic enables CB composites to reach the percolation threshold at lower loading rates than CF composites [43,44]. For wood-based composites, this phenomenon has not yet been described.

Adding 5 wt.% CB transforms the wood-based composite from insulators to semiconductors. When the CB content reaches 10 wt.%, a significant decrease in electrical resistance is observed. This phenomenon is associated with the percolation threshold, the specific concentration at which conductive materials create a continuous network across the matrix. This leads to a marked shift from an insulating to a conducting state. At this threshold, the filler particles come into such proximity that they create an interconnected network, enabling the matrix material’s dielectric breakdown [45].

As a result, there is an immediate and substantial decrease in electrical resistivity at the percolation threshold as electrons are able to efficiently move through the filled wood matrix via hopping or quantum tunneling [45]. The filler creates a percolation network that adds electrical conductivity to an otherwise insulating matrix because of the significant difference in electrical conductivity between the filler and the matrix [44].

Interestingly, in contrast to the nano-sized CB, adding CF at low levels (5–10 wt.%) did not result in any electrical conductivity. The findings indicate that the wood/CF percolation threshold was only achieved at a higher CF loading level of 20 wt.%. This suggests that polymer composites containing CB exhibit a lower percolation threshold due to their higher surface area, similar to findings by Motlagh et al. on carbon-filled olefin composites [43]. Consequently, a more significant number of CFs is required to achieve a conductive composite compared to CB systems [44].

The percolation threshold may also noticeably shift in favor of a lower CB content due to improved dispersion techniques. The dispersion state of CB and CFs is crucial in determining the performance, particularly the electrical properties, of composites filled with these carbon materials. Enhanced dispersion ensures that the conductive pathways are more efficiently established, thereby optimizing the composite’s electrical conductivity, as it is known for polypropylene composites [42].

The tunneling effect in carbon-based materials enhances the electrical conductivity of polymer composites. Conductivity arises not only through direct contact between fillers but also via electron tunneling when the distance between adjacent carbon particles is small enough. This quantum tunneling allows electrons to move through insulating gaps, forming conductive pathways. Factors, such as filler concentration, dispersion, particle size, and polymer–filler interactions influence the extent of this effect [46,47].

**Figure 7:**
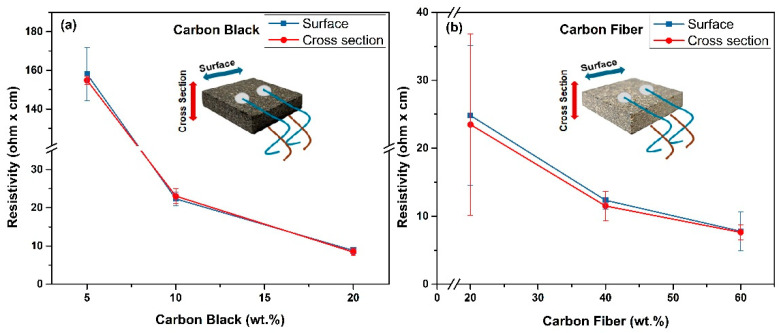
Surface and cross-section electrical resistivity measurement (**a**) CB samples and (**b**) CF samples.

Figure 7 shows the electrical resistivity measurements taken from the surface and cross-sectional area. The measurements were made by taking ten measurements from the surface and cross-sectional areas of five different samples, and the mean and standard deviation of the values were calculated. It is expected, according to findings on fiberboards [20], that 2-dimensional conductive networks on the surface are observed at lower CF contents than a 3-dimensional conductive network.

For the CB5 sample in Figure 7a, almost identical results are obtained for the cross-sectional areas, while the surface measurements exhibit a standard deviation of about 20%. The CB10 and CB20 samples showed consistent results across all measurements.

In the case of the CF20 sample in Figure 7b, the standard deviation for surface measurements is 30%, and for cross-sectional measurements, it is 40%. A standard deviation of approximately 10% and 20% was calculated for the CF40 and CF60 samples, respectively.

When these results are examined, it is further evident that different measurements yield varying outcomes depending on their location, the filler distribution, and network homogeneity. Additionally, CB is more homogeneously distributed in the matrix than CFs. The SEM images support these findings. Consequently, more homogeneously dispersed CB composites are expected to reach the percolation threshold at lower rates than CFs composites, resulting in lower electrical resistance values.

### 3.3. Moisture Effect on Resistance

**Figure 8:**
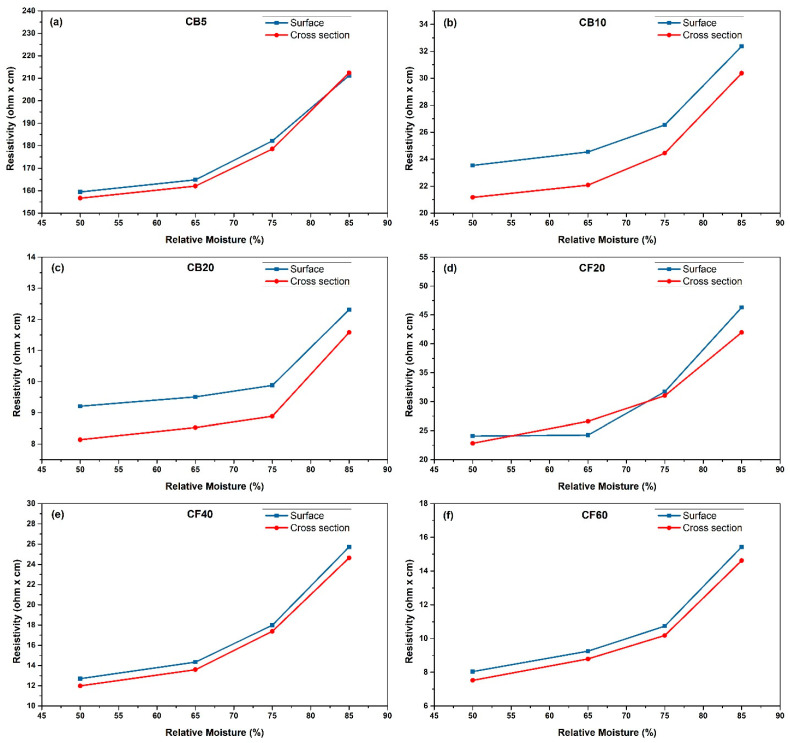
Moisture effect on electrical resistivity, (**a**) CB5; (**b**) CB10; (**c**) CB20; (**d**) CF20; (**e**) CF40 and (**f**) CF60.

The resistance of the conductive PBs was measured under varying moisture conditions. All results are shown in Figure 8. For each tested sample, an increase in moisture content resulted in higher resistance values (Figure 8a–f). These results indicate that moisture adversely affects the electrical conductivity of the PBs by increasing their resistance.

**Figure 9:**
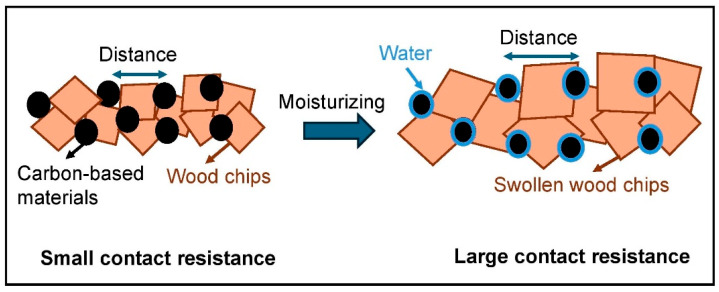
The effect of water on electrical contact resistance in particle boards.

This behavior can be attributed to water adsorption increasing the contact resistance of carbon-based materials, thereby reducing electrical conduction, as described by Zhang et al. for CB-filled cement-based materials [48]. According to this theory, the moisture-adsorbing wood particles effectively become a two-phase composite with higher resistivity due to the moisture and lower resistivity due to the filler. Consequently, higher moisture content results in more excellent contact resistance. Table 2 shows the resistance values of all samples before drying in the climate chamber, at different moisture conditions and after drying. When the swollen wood particles were dried, the resistance and thickness values of the composites decreased again, but not mainly to their original levels. This can again be explained using the theory of Zhang et al.: due to the moisture treatment, the distances between the carbon particles could possibly remain increased, even after a complete drying process [48]. This dependence on moisture can be in addition explained by a swelling behavior of the wood particles in high moisture conditions, described by Park et al. for CNT-filled agarose composites [49]. As the wood particles swell, the thickness of the composites increases, leading to a greater distance between the conductive fillers and a reduction in the number of filler junctions (Figure 9). This effect results in an increased resistance.

The positive dependence on moisture is generally attributed to the swelling behavior of the polymer matrix. When exposed to high moisture levels, the hydrophilic polymer matrix swells, causing an increase in the distance between the conductive fillers. This increased distance reduces the number of filler connections, leading to higher resistance. This swelling behavior disrupts the conductive pathways formed by the fillers, resulting in a significant increase in resistance [50,51,52].

For the PBs, Figure 9 presents this theory of how CB and CFs can show a positive moisture dependence of the resistance in the composite, explained by the wood’s swelling behavior.

### 3.4. Temperature Effect on Resistance

**Figure 10:**
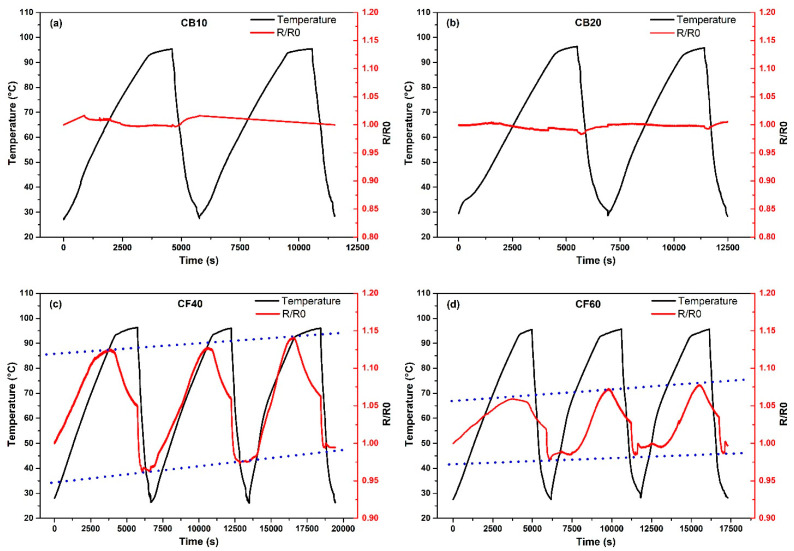
Temperature effect on electrical resistivity, (**a**) CB10; (**b**) CB20; (**c**) CF40; and (**d**) CF60.

Measurements of the resistance change were monitored during the temperature change at a constant moisture. To test the sensor properties of the PBs, three consecutive heating–cooling cycles were performed. When the results were analyzed, only the sample CF40 (Figure 10c) showed reproducible values and a positive temperature coefficient (PTC). In contrast, in the case of the CB10 and CB20 (Figure 10a,b) samples, there was only a slight change in the resistance values, which remained almost constant. This constant behavior of the CB samples can be helpful to use these samples on the other hand specifically as a moisture or pressure sensor.

According to Kim et al., CB-loaded nanocomposites have been attracting increasing attention as PTC materials [53]. However, for the measured CB-containing wood samples in the present study, resistance values remained almost unchanged (see Figure 10a,b). Thus, the CB10 and CB20 samples behave like zero-TCR composites. In many electronic device applications, resistors with a small or zero TCR are desirable. This is especially important to ensure stability and reliability [54].

The reason for this zero TCR behavior is explained by Liu et al. [55]: a more robust filler conductor network reduces distortion due to the thermal expansion of the polymer matrix and minimizes or eliminates the PTC effect. These robust networks can result from smaller filler sizes, higher aspect ratios, and improved filler dispersion. The multiplicity of contact points combined with highly interconnected or entangled meshes can lead to a lack of PTC or NTC effect as the temperature increases.

Although CB composites cannot be used as temperature sensors in this study, they can be used for applications where precise and stable control of temperature is required.

The observed increase in Figure 10c,d in electrical resistance with rising temperature in wood–CF composites follows the behavior of PTC materials, which can be explained to some extent by thermal expansion as described in previous studies [56,57,58]. Similar PTC behavior as monitored in Figure 10 was presented in [56], where a slow increase in resistivity at the beginning to the heating was shown, followed by a faster increase and the reaching of a maximum. However, in contrast to these samples, the effect measured in our present study on wood-based panels cannot be attributed to the thermoplastic matrix. The effect is rather attributed to the expanding wood particles.

Conductive polymer composites showing a PTC are semi-crystalline polymers with conducting filler concentrations close to the critical volume fraction [59]. Several researchers have established that conductive polymer composites with larger average filler sizes exhibit higher PTC density and greater resistance at room temperature compared to systems with smaller filler sizes, even at similar filler content [60,61].

This phenomenon is likely associated with the increase in average interparticle distance accompanying the larger particle size. In addition, as the size of the filler decreases, the proportion of filler used increases, and/or more robust conductive pathways are obtained in the matrix [55,61]; it can be seen that the PTC density decreases with temperature or exhibits NTC behavior [55].

The PTC behavior of conductive composites depends on the “robustness” of the conductive network formed within the polymer matrix. Therefore, the conductive network in conductive polymer composites can be considered the primary factor influencing the PTC effect, encompassing contributions from both the filler and the matrix [55,61,62]. Several theories have already been proposed to explain the PTC mechanism [57]. However, the exact mechanism of the PTC behaviors of CB composites is an unsolved issue [58].

All measured CF samples (Figure 10c,d) showed a sharp decrease of the resistance at around 85–90 °C, which can be related to water vaporization from the moisture being present inside the wood chips. This water vaporization results in highly conductive polar ions present also inside the wooden composite and probably increasing the conductivity. With this decrease, the samples demonstrate a NTC effect above 90 °C.

After each cycle, the baseline of the electrical resistance values shows an increasing trend compared to the initial values. This increase of the baseline is possibly a result of the degradation of the wood structure that occurs with each heating and cooling cycle.

### 3.5. Pressure Effect on Resistance

**Figure 11:**
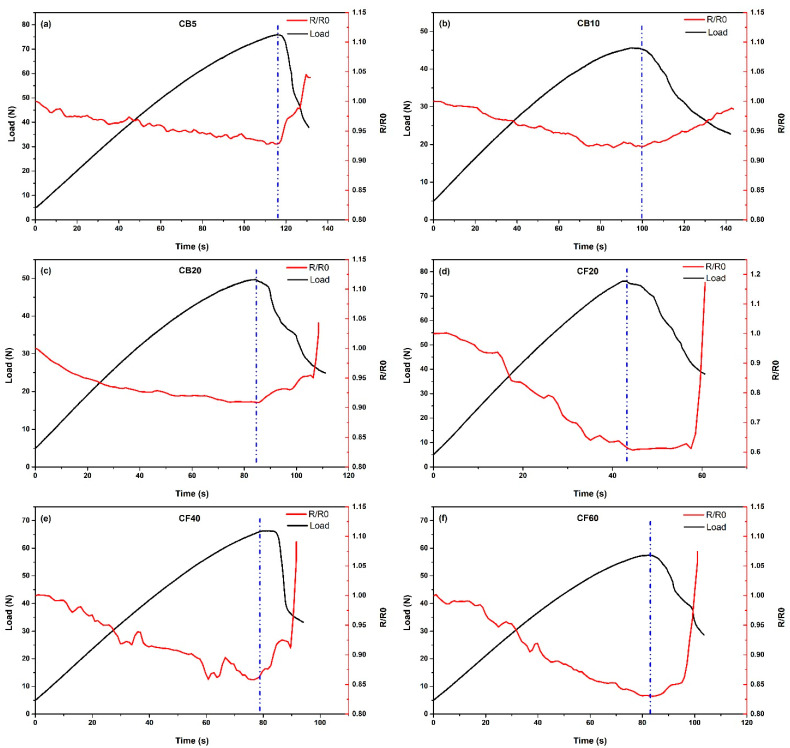
3-point bending test with time-dependent resistance change, (**a**) CB5; (**b**) CB10; (**c**) CB20; (**d**) CF20; (**e**) CF40; and (**f**) CF60.

Pressure sensor properties or potential human movement detection of the samples were examined using a 3-point bending test, and the results are presented in Figure 11. The findings indicate that resistance values decrease in all samples as the loading increases. The blue line on the graph indicates the moment when the break in the PB begins. This point marks the transition where the material starts to crack, resulting in an increase in resistance as gaps form and expand within the composite structure. This behavior aligns with the literature, which describes the percolation network as being composed of both permanent and transient structures [63,64,65].

**Figure 12:**
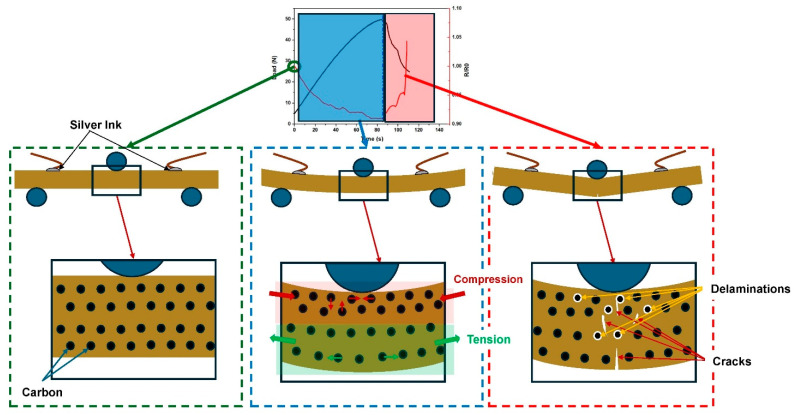
Demonstration of the change in electrical resistance under pressure.

The observed decrease in resistance during loading can be attributed to the different compressibility coefficients of the rigid CB and CF fillers compared to the porous wood particles. The distance between the carbon materials decreases under load, which, in turn, creates additional conductive pathways. Although some carbon chains may be disrupted due to sliding or reorganization under pressure, this damage is not seen during loading [65]. As shown in Figure 12, under the influence of pressure, the carbon particles move closer to each other, leading to a decrease in electrical resistance. However, as soon as cracks begin to form in the PB, the resistance values begin to rise again.

The CB samples showed a variation between 7% and 10% in the resistance due to the change in loading before breaking. In contrast, the CF samples exhibited a change in resistance ranging from 15% to 40%. Notably, CF20 demonstrated the greatest sensitivity to pressure, with a change of 40%. This increased sensitivity can be attributed to the CF’s fiber structure, which allows the fibers to come into closer contact with each other under compression, unlike the spherical structure of CB. As a result, changes in resistance values are more pronounced and observable in CF.

The load value of 75 N in the CB5 sample decreased to 50 N in the CB20 sample. Similarly, the load value of 75 N in the CF20 sample decreased to 65 N in the CF60 sample. This reduction in load-bearing capacity with increasing carbon content can be attributed to the weakening of the composite’s mechanical properties due to higher carbon filler concentrations, which likely result in agglomeration and stress concentration points. This decrease in flexural stress may also be due to inhomogeneous distribution of carbon materials.

In this way, it is possible to detect human movement based on changes in pressure or to monitor any damage to the PB. The variation in resistance due to pressure changes can serve as an indicator both for sensing human movement and also for identifying structural integrity issues within the PB.

### 3.6. Mechanical Properties

**Figure 13:**
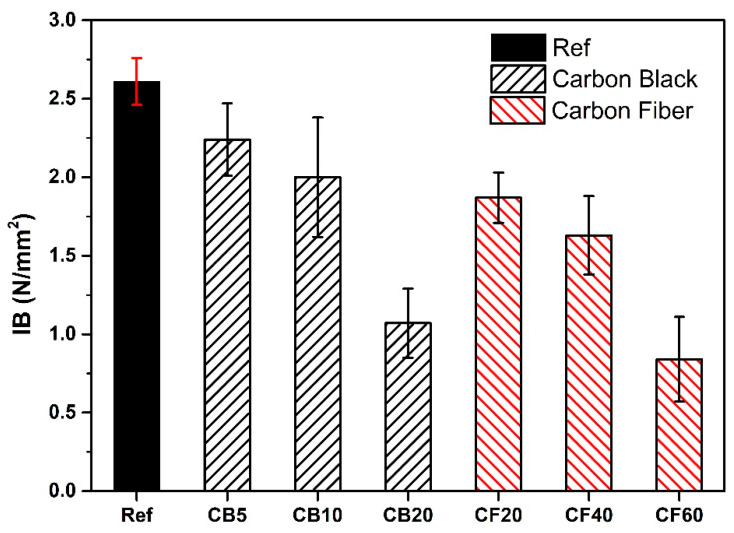
IB results of the particle boards.

The mechanical properties of conductive PBs were evaluated using the EN 319 standard, which requires a minimum tensile strength of 0.40 N/mm^2^. The results shown in Figure 13 indicate that all PBs met this standard. However, the reference sample exhibited the highest tensile strength. A decline in mechanical properties was observed with the addition of carbon. This can be attributed to several factors. Firstly, carbon fillers tend to agglomerate within the matrix due to their inhomogeneous distribution, creating stress points that weaken the material [66]. Additionally, these fillers are less compatible with the adhesive compared to the wood chips, leading to poor interfacial interaction and adhesion. As the concentration of carbon-based fillers increases, the tensile strength further diminishes [20,21,67].

In summary, CF-containing composites show better mechanical properties than CB-containing composites, even though they contain a higher percentage of filler by weight. Studies in the literature indicate that CFs exhibit superior mechanical properties compared to CB [68,69]. Additionally, it has been demonstrated for several other composites that CFs generally enhance the mechanical properties of composites [70,71,72].

## 4. Discussion

The resulting conductive and moisture-, pressure-, or temperature-sensitive panels could be designed in future applied studies to be easily incorporated into other wooden composite products in accordance with the “smart house” concept. Temperature and moisture sensors could be strategically placed at key points such as kitchen or bathroom entrances, which are expected to respond quickly to adverse situations like flooding or fire. Pressure sensors can be installed at home entrances to provide rapid responses in unexpected, suspicious, or adverse conditions, such as detecting the entry of a burglar. These sensors can enhance home security by identifying pressure changes and triggering alerts in real-time. However, for industrial applications, further optimization is required. Based on the results of the current study, a more detailed analysis of the percolation threshold should be performed in order to evaluate mixtures with more pronounced and reproducible sensing capabilities and to improve the economic viability of the materials by reducing the filler content. Exploring the synergistic effect of using mixed carbon materials could also help reduce the amount of carbon used. In addition, optimizing the mixing technique of the wooden panels is required to enhance the sensor panel homogeneity. The cyclic behavior of the moisture resistance changes of the samples (moisturize–dry–moisturize–dry) will be further investigated in future studies.

Studies comparing the electrical resistivity values of wood-based panels are presented in Table 3. Upon reviewing the literature, it becomes evident that this study differs from previous research. While the literature predominantly focuses on enhancing the electrical conductivity of wood-based panels, the current study emphasizes improving electrical conductivity and developing these panels’ sensor properties. This approach introduces new functionalities, such as temperature, moisture, and pressure sensing, expanding the potential applications of wood-based panels in smart systems.

## 5. Conclusions

This study aimed to develop electrically conductive materials to be used as sensors to detect moisture, water uptake, increasing temperature, or human movement detection via pressure change. For this purpose, single-layer PBs were produced by hot pressing using CB and CFs as conductive elements. The PBs could be electrically conductive with additives and different mixing ratios. Although the percolation threshold is achieved in the range of 5–10% CB by weight, it requires the addition of 20 wt.% CFs to reach the percolation threshold. The electrically conductive panels were examined regarding electrical properties under constant and varied environmental conditions, such as moisture, temperature, and pressure. PBs produced with both additive types could be used as moisture and pressure sensors, while CF filler was found to be potentially applicable for use as a temperature sensor, too. Significantly, the sample containing 40 wt.% CFs demonstrated a reproducible PTC. All conductive PBs exhibited changes in electrical resistivity in response to varying pressure. Additionally, composites containing CFs were found to be more sensitive to pressure than those containing CB.

The mechanical properties of the produced PBs were evaluated using the internal bond test. Despite decreasing strength upon filler addition, the mechanical properties of all the samples met the standard requirements for PBs.

## Figures and Tables

**Table 1:** Recipe and identification of the wood composites.

Sample Code	Filler Type	Filler Ratio (%)	Wood Chips Ratio (%)	Resin Ratio (%)
Ref	-	0	90	10
CB5	Carbon black	5	85	10
CB10	Carbon black	10	80	10
CB20	Carbon black	20	70	10
CF20	Carbon fiber	20	70	10
CF40	Carbon fiber	40	50	10
CF60	Carbon fiber	60	30	10

**Table 2:** Change of resistance values of the samples in dry and different relative moisture conditions.

Sample Code	Before RM	50% RM	65% RM	75% RM	85% RM	Dry	Measurement Place
CB5	158.3	159.4	164.8	182.1	211.2	184.8	Surface
	155.2	156.7	162.1	178.5	212.4	185.4	Cross-section
CB10	23.7	23.5	24.5	26.5	32.4	26.8	Surface
	20.9	21.2	22.1	24.4	30.4	24.5	Cross-section
CB20	8.8	9.2	9.5	9.9	12.3	9.1	Surface
	8.0	8.1	8.5	8.9	11.6	10.1	Cross-section
CF20	23.4	24.1	24.2	31.7	46.3	22.1	Surface
	22.0	22.8	26.6	31.0	42.0	20.7	Cross-section
CF40	12.7	12.8	14.3	18.0	25.7	16.6	Surface
	11.6	12.0	13.6	17.4	24.6	15.6	Cross-section
CF60	8.0	8.0	9.2	10.7	15.4	11.2	Surface
	7.5	7.5	8.8	10.2	14.6	10.5	Cross-section

**Table 3:** Comparison of electrical resistivity results previously reported in the literature with the present study.

Panel Type	Filler Type	Resin Type	Resistivity (ohm.cm)	Additional Outcomes	Sensor Properties	Ref.
PB	Carbon nets	UF	0.14–0.58	0.5 and 2 mm yarn width	-	[2]
MDF	CFs	UF and pMDI	5–16	3 mm CF lenght	-	[19]
MDF	CFs	pMDI	9–50	10 mm CF lenght	-	[20]
MDF	CFs	UF and pMDI	-	6 mm CF lenght EMI-SE: 23–64 dB	-	[21]
MDF	CFs	UF and pMDI	0.4–322	3 mm CF lenght	-	[22]
PB	CFs& CB	pMDI	4.8–179	CF: 100–400 µm CB: 50 nm	Moisture Temperature Pressure	Present study

## Data Availability

The data presented in this study are available on request from the corresponding author.
